# A Mechanism for Statin-Induced Susceptibility to Myopathy

**DOI:** 10.1016/j.jacbts.2019.03.012

**Published:** 2019-08-26

**Authors:** Sabine Lotteau, Niklas Ivarsson, Zhaokang Yang, Damien Restagno, John Colyer, Philip Hopkins, Andrew Weightman, Koichi Himori, Takashi Yamada, Joseph Bruton, Derek Steele, Håkan Westerblad, Sarah Calaghan

**Affiliations:** a School of Biomedical Sciences, Faculty of Biological Sciences, University of Leeds, Leeds, United Kingdom; b Department of Physiology and Pharmacology, Karolinska Institutet, Stockholm, Sweden; c VetAgro Sup, APCSe, Université de Lyon, Marcy l’Etoile, France; d Leeds Institute of Medical Research at St James’s, University of Leeds, Leeds, United Kingdom; e School of Mechanical, Aerospace and Civil Engineering, University of Manchester, Manchester, United Kingdom; f Graduate School of Health Sciences, Sapporo Medical University, Chuo-ku, Sapporo, Japan

**Keywords:** calcium leak, exercise, myopathy, ryanodine receptor, statin, Ca^2+^, calcium, FDB, flexor digitorum brevis, FKBP12, FK506 binding protein (calstabin), GAS, gastrocnemius, HADHA, hydroxyacyl-CoA dehydrogenase/3-ketoacyl-CoA thiolase/enoyl-CoA hydratase, HMG CoA, 3-hydroxy-3-methylglutaryl coenzyme A, L-NAME, N(ω)-nitro-L-arginine methyl ester, NOS, nitric oxide synthase, PGC1α, peroxisome proliferator-activated receptor γ co-activator 1α, RNS, reactive nitrogen species, ROS, reactive oxygen species, RyR, ryanodine receptor, SOD, superoxide dismutase, SR, sarcoplasmic reticulum, TUNEL, terminal deoxynucleotidyl transferase dUTP nick end labeling

## Abstract

This study aimed to identify a mechanism for statin-induced myopathy that explains its prevalence and selectivity for skeletal muscle, and to understand its interaction with moderate exercise. Statin-associated adverse muscle symptoms reduce adherence to statin therapy; this limits the effectiveness of statins in reducing cardiovascular risk. The issue is further compounded by perceived interactions between statin treatment and exercise. This study examined muscles from individuals taking statins and rats treated with statins for 4 weeks. In skeletal muscle, statin treatment caused dissociation of the stabilizing protein FK506 binding protein (FKBP12) from the sarcoplasmic reticulum (SR) calcium (Ca^2+^) release channel, the ryanodine receptor 1, which was associated with pro-apoptotic signaling and reactive nitrogen species/reactive oxygen species (RNS/ROS)−dependent spontaneous SR Ca^2+^ release events (Ca^2+^ sparks). Statin treatment had no effect on Ca^2+^ spark frequency in cardiac myocytes. Despite potentially deleterious effects of statins on skeletal muscle, there was no impact on force production or SR Ca^2+^ release in electrically stimulated muscle fibers. Statin-treated rats with access to a running wheel ran further than control rats; this exercise normalized FKBP12 binding to ryanodine receptor 1, preventing the increase in Ca^2+^ sparks and pro-apoptotic signaling. Statin-mediated RNS/ROS−dependent destabilization of SR Ca^2+^ handling has the potential to initiate skeletal (but not cardiac) myopathy in susceptible individuals. Importantly, although exercise increases RNS/ROS, it did not trigger deleterious statin effects on skeletal muscle. Indeed, our results indicate that moderate exercise might benefit individuals who take statins.

## Highlights

• The authors used human and rat muscle to study the mechanism of statin myopathy and its interaction with exercise.
• Statin treatment triggered loss of the modulator protein FKBP from the sarcoplasmic reticulum (SR) calcium (Ca^2+^) release channel, ryanodine receptor 1 (RyR1).
• Loss of FKBP was associated with reactive nitrogen species/reactive oxygen species-dependent SR Ca^2+^ leak and pro-apoptotic signaling, but had no overt impact on muscle function.
• Moderate running wheel exercise prevented the effects of statin treatment on the FKBP/RyR complex, SR Ca^2+^ leak, and pro-apoptotic signaling.
• Our data show that statin treatment induces a potentially harmful SR Ca^2+^ leak that might trigger statin myopathy in susceptible individuals, but could be prevented by moderate exercise.

## Visual Abstract

**Figure:**
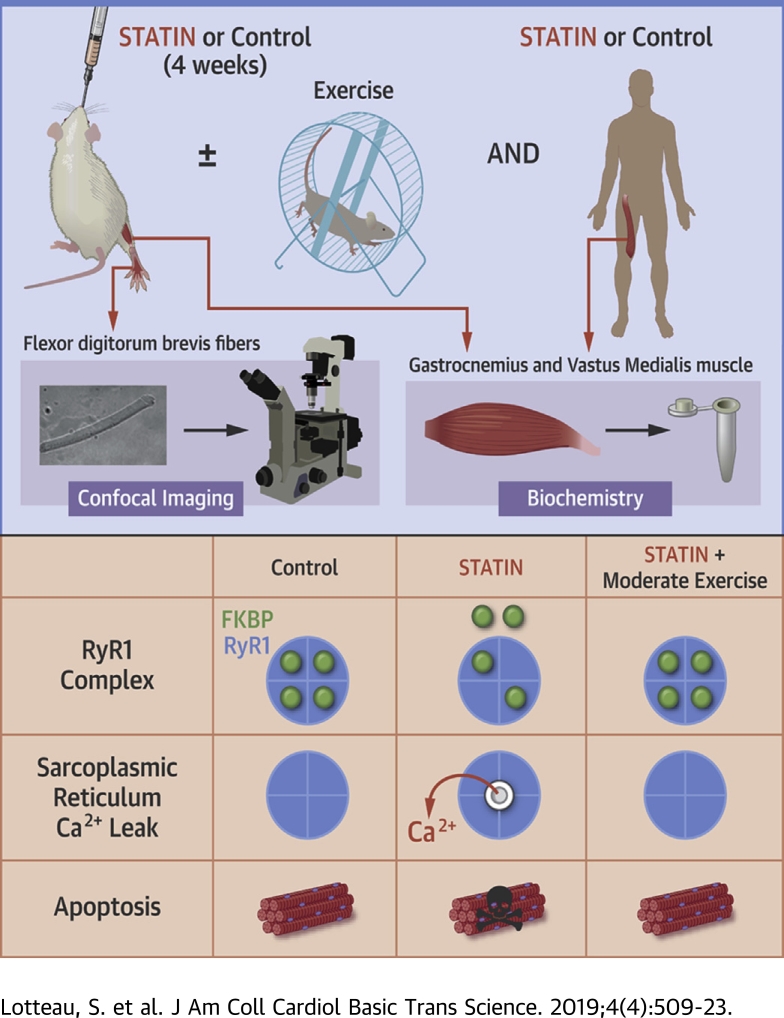

Statins are the most widely prescribed drug in the Western world. Their use is predicted to rise further due to recent reductions in the cardiovascular risk threshold for statin prescription across the globe 1, 2. However, cardiovascular benefits of statins are restricted by adverse effects that limit adherence 3, 4 and, in turn, increase cardiovascular events (5) and mortality (6). The most common side effects and main reason for discontinuation of therapy emerge from skeletal muscle (statin myopathy or statin-associated adverse muscle symptoms). Although no strict definition of statin myopathy has been universally adopted 7, 8, 9, 10, we use this term to encompass the full spectrum of the effects of statins on skeletal muscle. This includes mild to moderate muscle symptoms and/or signs (myalgia: muscle pain with stiffness and weakness), as well as more severe potentially life-threatening outcomes (myositis and/or rhabdomyolysis) that are associated with raised creatine kinase 8, 11. Although physical activity counteracts metabolic and cardiovascular diseases that are prevalent in subjects prescribed statins, exercise has been reported to exacerbate statin myopathy 12, 13, 14, 15, 16, 17, 18, 19, which may further limit the benefits of statins in those at risk of cardiovascular disease.

Statins are inhibitors of 3-hydroxy-3-methylglutaryl coenzyme A (HMG CoA) reductase that limit the production of cholesterol, isoprenoids, and coenzyme Q. Despite extensive research, which has focused on calcium (Ca^2+^) homeostasis and mitochondrial function 20, 21, 22, 23, 24, 25, a cohesive mechanism for statin-induced myopathy is lacking. Furthermore, an understanding of why myopathy is not experienced by everyone who takes statins and the reason for its selectivity for skeletal muscle has not been fully addressed.

Using human and rodent muscle, we investigated the mechanism for statin-induced myopathy and described its interaction with voluntary moderate exercise. We revealed a mechanism by which statin treatment can make skeletal muscles susceptible to myopathy—dissociation of the FK506 binding protein (FKBP12) from the sarcoplasmic reticulum (SR) Ca^2+^ release channel, the ryanodine receptor 1 (RyR1), which is accompanied by numerous spontaneous Ca^2+^ release events (i.e., Ca^2+^ sparks) (26). Statin treatment had no effect on Ca^2+^ sparks in cardiac muscle. Aberrant SR Ca^2+^ handling was associated with pro-apoptotic signaling in skeletal muscle. However, despite this myopathy-promoting signaling, statin treatment had no obvious detrimental effect on the contractile function of skeletal muscle, which suggests that additional factors are required to produce myopathic symptoms. Furthermore, in rats that underwent voluntary exercise, no overt muscle dysfunction was evident. Our data demonstrate that individuals taking statins might benefit from moderate exercise.

## Methods

### Study approval

Anonymized vastus medialis samples were obtained from patients who were screened (and tested negative) for malignant hyperthermia. Individuals taking statins were age- and sex-matched with control subjects (Table 1). All patients gave informed consent. This study complied with the principles of the Declaration of Helsinki and was approved by the Leeds East Local Research Ethics Committee. Work with rodents was performed in strict accordance with the recommendations of the Directive 2010/63/EU of the European Parliament and was approved by animal welfare committees at the University of Leeds and the Karolinska Institutet.

**Table 1:** Patient Data

Statin			Statin				Matched Control Subjects			
	Dose (mg)	Sex	Age (yrs)	Histology	CK (IU/l)	Disease	Age (yrs)	Histology	CK (IU/l)	Disease
SIMV	40	F	48	Type 2b fiber atrophy		HC, H	47	Normal		Treated hypothyroidism
SIMV	40	M	72	Type 2b fiber atrophy		RM	73	Type 2b fiber atrophy	110	H, DM, minor CVA
SIMV	20	M	65	Fiber size, variation, increase in mitochondria	76	H, RM	65	Type 2b fiber atrophy	200	
SIMV	40	F	60	Normal		Type 2 DM, CVA, obese	60	Normal	110	
SIMV	40	M	70	Normal		H, AA	70	Normal	142	H
SIMV	10	M	71		122	H	71	Fiber size variation		H, CVA
SIMV	40	M	48	Normal	157	H	48			
PRAV	30	M	58	Atrophy in scattered fibers		H	58	Normal	102	
SIMV	20	M	72	Atrophy and angulation in many fibers	116	H	71	Normal	138	MV
SIMV	40	M	59			Type 2 DM, CVA, obese	59	Normal	97	
ATOR	20	M	56			IHD	56	Normal		H
ROSU	10	F	54	Normal	114	H	54			
SIMV	20	F	52	Normal	57	H	51	Normal		

All samples from patients taking statins were paired with sex- and age-matched control subjects.

Details of histology, serum creatine kinase (CK), and disease are given where available.

AA = aortic aneurysm; CVA = cerebrovascular accident; DM = diabetes mellitus; H = hypertension; HC = high cholesterol; IHD = ischemic heart disease; MV = mitral valve disease; RM = risk modification.

### Rodent models

Male Wistar rats (130 to 160 g) received simvastatin (40 mg/kg/day) or saline by oral gavage at the beginning of the dark cycle for 28 days (see Supplemental Methods for justification of dose). For exercise studies, rats were given free access to an in-cage running wheel. Custom-built hardware and software allowed detailed characteristics of running activity to be recorded for each animal (see Supplemental Methods). All animals were killed by stunning and cervical dislocation.

### Rodent muscle Preparations

For protein chemistry, rat gastrocnemius (GAS) muscle (a predominantly type II muscle) was dissected to remove slow oxidative type I fibers (dark red in color). For confocal microscopy, rat flexor digitorum brevis (FDB) fibers were isolated by collagenase digestion (27). FDB is predominantly type IIa; the choice of this muscle was informed by the short length of the fibers that allows the isolation and study of intact cells. In some cases, fibers were permeabilized by 2-min exposure to 0.005% (w/v) saponin (28). Rat cardiac myocytes were isolated from Langendorff-perfused hearts by collagenase and protease digestion (29).

### Muscle function in vitro

Rat single FDB fibers were dissected, mounted, and electrically stimulated via platinum plates. The isolated muscle preparations were stimulated for 350 ms at 10 to 150 Hz at 1-min intervals, and the resultant force was measured. The fluorescent Ca^2+^ indicator indo-1 was pressure injected into fibers, and the fluorescence signals of indo-1 were recorded at rest and during contractions as described previously (30).

### Confocal microscopy

Confocal images were acquired with a Eclipse TE300 inverted microscope (Nikon, Minato, Tokyo, Japan) equipped with a confocal scanhead, MicroRadiance 2000 (Bio-Rad, Hercules, California), and a ×60 water-immersion objective. FDB fibers were loaded with fluo 4-AM (5 μM, for intact cells), fluo 3 (50 μM, for permeabilized cells), or DAF-2 (5 μM). Cardiac myocytes were loaded with fluo 4-AM (6 μM). Dyes were excited with the 488-nm line of a 20-mW coherent sapphire laser (attenuated ≈90%), and emitted fluorescence was measured at >515 nm. Images were acquired in x-y (every 5 s) or line scan mode (every 6 ms). Ca^2+^ sparks were identified and analyzed with ImageJ software version 1.51j8, National Institutes of Health, Bethesda, Maryland) using the Sparkmaster plugin (see Supplemental Methods).

### Protein chemistry and assays

Sodium dodecyl sulfate-polyacrylamide gel electrophoresis and Western blotting were carried out as described in Calaghan et al. (29). Data were normalized to glyceraldehyde-3-phosphate dehydrogenase (GAPDH) expression. Because it was not possible to load all samples on the same gel, a standard calibration sample (mixed from 4 human vastus medialis samples or 3 rat GAS samples) was loaded in duplicate on gels to allow between-gel comparisons. For RyR post-translational modifications and protein associations, RyR1 was immunoprecipitated from GAS as described previously (31) (see Supplemental Methods). The terminal deoxynucleotidyl transferase dUTP nick end labeling (TUNEL) assay was performed on cryostat sections (10 μm; Leica CM 1900), visualized using the detection kit TACS 2TdT-DAB for In situ Apoptosis (4810-30-K, Trevigen) (32). Calpain activity was assessed using the assay kit QIA120 (Merck Millipore).

### Antibodies

Antibodies were as follows: calmodulin Abcam Cat# ab45689 RRID:AB_725815, 1:1,000; FKBP12 Abcam Cat# ab58072 RRID:AB_941602, 1:200; hydroxyacyl-CoA dehydrogenase/3-ketoacyl-CoA thiolase/enoyl-CoA hydratase (HADHA) Abcam Cat# RRID:AB_2263836 1:1,000; peroxisome proliferator-activated receptor γ co-activator 1α (PGC1α) Abcam Cat# ab54481 RRID:AB_881987, 1:1,000; RyR clone 34C Abcam Cat# ab2868 RRID:AB_2183051, 1:5,000; Cav 3 BD Biosciences Cat# 610420 RRID:AB_397800, 1:5000; endothelial nitric oxygen synthase eNOS BD Biosciences Cat# 610297 RRID:AB_397691, 1:2,500; Cav 1 Boster Biological Technology Cat# PA1514, RRID: AB_2651038, 1:1,000; caspase-3 Cell Signaling Technology Cat# 9665 also 9665S RRID:AB_2069872, 1:1000; nNOS Cell Signaling Technology Cat# 4231S RRID:AB_2152485, 1:1,000; and glyceraldehyde-3-phosphate dehydrogenase (GAPDH) Sigma-Aldrich Cat# G9545 RRID:AB_796208, 1:100,000.

### Statistical analysis

Results are expressed as mean ± SEM of number of observations, with p < 0.05 used to denote statistical significance. The Shapiro-Wilk test was used to test for normality. For the human study, we had access to 13 samples from statin-treated individuals and 13 age- and sex-matched control subjects. The paired Student’s *t*-test (normally distributed data) or the Wilcoxon signed-rank test (non-normally distributed data) were used to compare groups. This sample size gave power >0.8 to detect a 100% change in parameter (SD: 100% of mean; paired Student’s *t*-test). For rodent samples, comparison of 2 groups was performed using the Student’s *t*-test (normal distribution) and the Mann-Whitney rank test (non-normal distribution). For tetanic and force Ca^2+^ measurements at different frequencies of stimulation, a 2-way repeated measures analysis of variance was used (with the Holm-Sidak post hoc test). Two-way analysis of variance (with the Tukey post hoc test) was used to analyze daily running distance with time in the control and statin groups, and the effect of exercise and statin treatment on markers of mitochondrial biogenesis. For rodent studies, we used 10/11 and 5/6 animals in the sedentary control/statin groups in the United Kingdom and Sweden (tetanic force and Ca^2+^ measurements), respectively. For the exercise study, we used 6/6 control/statin-treated animals. Group sizes were based on power calculations for protein chemistry data from the rat, which showed power >0.8 to detect a 50% difference in means when n = 6 (SD: 25% of mean; *t*-test). Presented data might have different numbers of animals for some endpoints due to sample limitations. GraphPad Prism (version 7.05, Graphpad, San Diego, California) was used for all statistical analysis, with the exception of tetanic force and Ca^2+^ measurements (Sigmaplot for Windows, version 13.0, Systat Software Inc., San Jose, California).

## Results

### Summary of experimental plan and key findings

Supplemental Table 1 provides a summary of all experiments performed, with key findings for both human and rodent preparations.

### Dissociation of FKBP12 from RyR1 and pro-apoptotic signaling in skeletal muscle of statin-treated humans and rats

**Figure 1:**
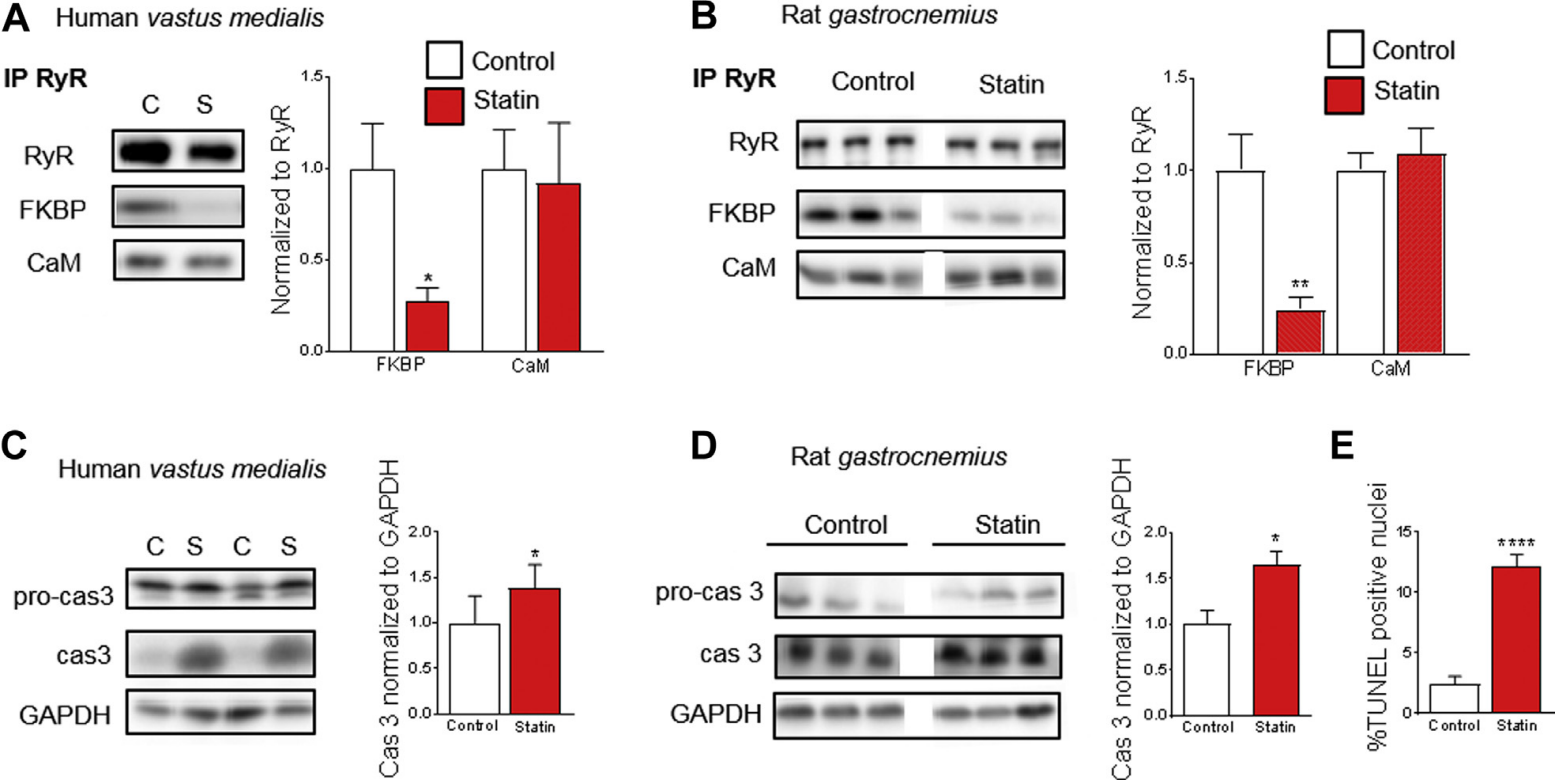
Dissociation of FKBP12 From RyR1 and Pro-Apoptotic Signaling in Skeletal Muscle From Statin-Treated Humans and Rats Representative blots from the same gel and mean data showing FK506 binding protein (FKBP12) and calmodulin (CaM) in ryanodine receptor (RyR) immunoprecipitates from **(A)** human and **(B)** rat muscle. All values are standardized to the mean of the control group. There was no difference (p > 0.05) in total RyR1 or FKBP12 expression between groups. Data from 11/11 (FKBP) and 13/13 (CaM) patients taking statins (S) and age- and sex-matched controls (C); n = 10 to 11 rats. **(C)** Representative blots from the same gel and mean data from human muscle showing pro-caspase 3 (pro-cas3; 35 kDa) and cleaved caspase 3 (cas3; 17 kDa). Data from 13/13 patients. **(D)** Expression of pro-cas3, cleaved cas3 and **(E)** proportion of terminal deoxynucleotidyl transferase dUTP nick end labeling (TUNEL) positive nuclei (%) in rat muscle. Cas3 expression is standardized to the mean of the control group. Data from 5 to 7 animals. All data are mean + SEM. **(A)** *p = 0.0127 (paired Student’s *t*-test). **(B)** **p = 0.0023 (Student’s *t*-test). **(C)** *p = 0.0425 (Wilcoxon signed-rank test). **(D)** *p = 0.0158 (Student’s *t*-test); ****p < 0.0001 (Student’s *t*-test). GADPH = glyceraldehyde-3-phosphate dehydrogenase.

Post-translational modifications of RyR1 and changes in the molecular composition of the RyR1 protein complex are present in several conditions with dysfunctional skeletal muscle 33, 34, 35. To test whether similar alterations occurred with statin treatment, we immuno-precipitated RyR1 in homogenates prepared from biopsies of human vastus medialis muscles and from isolated rat GAS muscles, and measured the expression of the RyR1 binding partners FK506 binding protein 12 (FKBP12) and calmodulin. Statin treatment caused a marked decrease in FKBP12 bound to RyR1 in both human and rat muscle, whereas the calmodulin binding remained intact (Figures 1A and 1B). It was noteworthy that a robust dissociation of FKBP12 from RyR1 could be detected, although muscle biopsies were obtained from a diverse patient group (Table 1).

FKBP12 dissociation from RyR1 has been shown to increase spontaneous SR Ca^2+^ leak, which, in turn, promotes protein degradation and programmed cell death (33). Therefore, we next studied whether the change in the RyR complex in muscles of statin-treated subjects was accompanied by indexes of pro-apoptotic signaling. For this purpose, we measured the protein expression of the inactive pro-caspase-3 and its cleaved active product, the pro-apoptotic enzyme caspase-3. Statin treatment increased caspase-3 expression in both human and rat muscles (Figures 1C and 1D). In rat muscle, we also measured the proportion of TUNEL positive nuclei, which is another marker for pro-apoptotic signaling, and observed a marked increase with statin treatment (Figure 1E). Thus, muscles from both humans and rats treated with statins showed major alterations that were potentially deleterious and might underlie statin-induced myopathy. In subsequent experiments, we delved deeper into mechanisms of the statin-induced effects; these experiments were only performed on rat muscles due to limitations in what can be performed on human muscle biopsy material.

### Statin treatment increases SR Ca^2+^ leak in intact skeletal muscle

**Figure 2:**
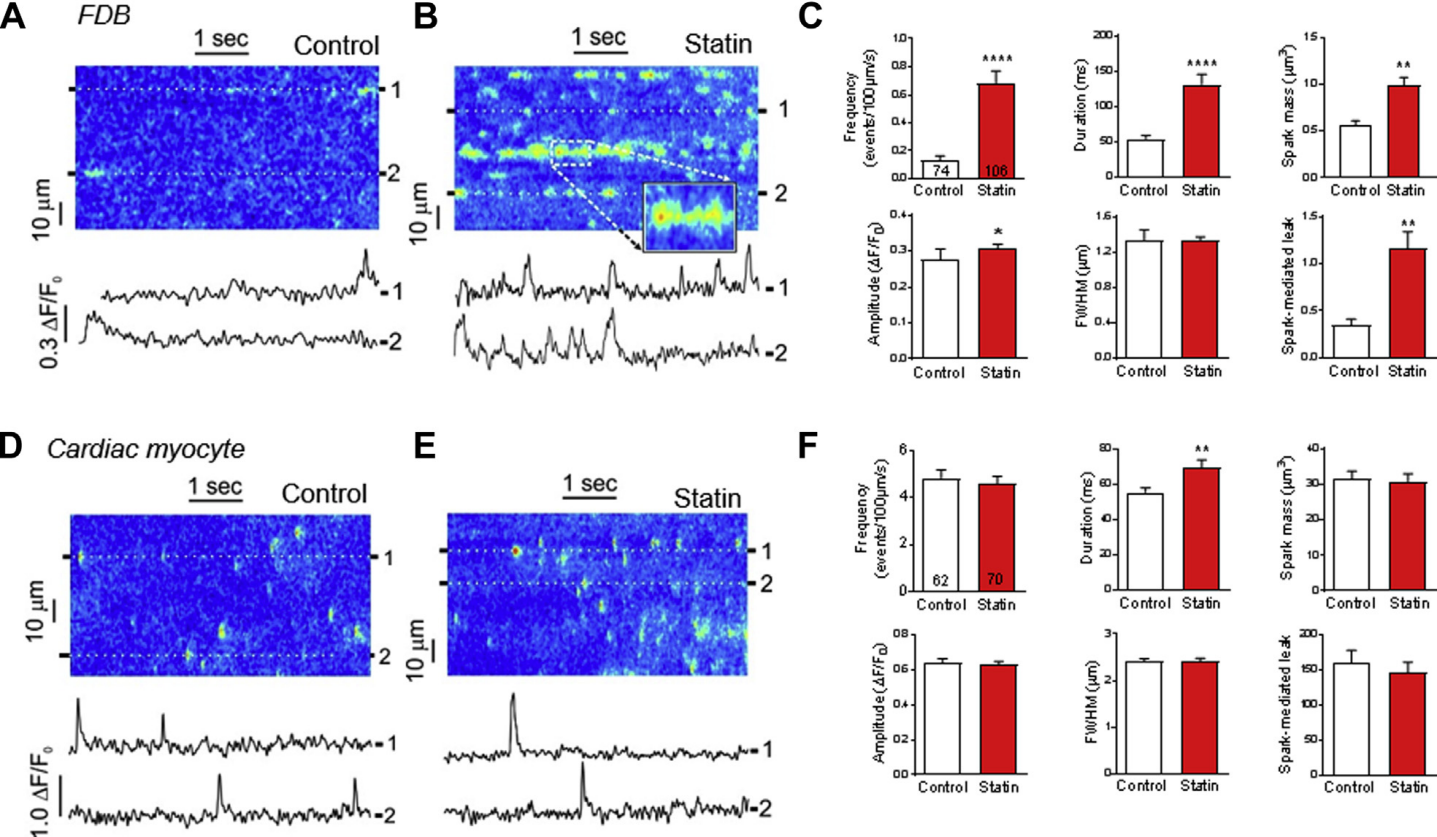
Statin Treatment Provokes SR Ca^2+^ Leak in Skeletal, But Not Cardiac, Myocytes Representative confocal line scans (F/F_0_) with associated line profiles and mean data from **(A to C)** intact flexor digitorum brevis (FDB) fibers and **(D to F)** cardiac myocytes; n = 11/10 (FDB) and 5/5 (cardiac myocytes) rats for control/statin-treated groups, number of cells shown on graphs. Data are mean + SEM and compared using the Mann-Whitney rank test. **(C)** ****p < 0.0001; *p = 0.0307; **p = 0.0029 (mass); **p = 0.0091 (leak). **(F)** **p = 0.0024. Ca^2+^ = calcium; FWHM = full width at half maximum; SR = sarcoplasmic reticulum.

SR Ca^2+^ leak in the form of Ca^2+^ sparks (elementary Ca^2+^ release events from clusters of RyR1) is a myopathic mechanism common to many skeletal muscle diseases, including muscular dystrophy and malignant hyperthermia 33, 36. Although spark-mediated SR Ca^2+^ leak is an attractive culprit for statin-induced myopathy, no overt changes in Ca^2+^ spark characteristics with (in vivo) statin treatment have been documented to date 20, 21, 23. However, all previous work has been performed on permeabilized muscle fibers, in which the constitutive inhibition of RyR1 by magnesium (37) and the dihydropyridine receptor (38) is reduced, which may mask the effects of statins. Therefore, we evaluated the effect of statin treatment on the SR Ca^2+^ leak in intact muscle fibers. Ca^2+^ sparks were recorded in nonpermeabilized fluo 4-loaded FDB fibers from the rat (Figures 2A to 2C). As predicted (39), spark frequency was low in intact fibers from control animals. In marked contrast, in fibers from statin-treated rats, sparks were much more frequent, of longer duration, and larger in amplitude, which resulted in an increased spark mass and spark-mediated Ca^2+^ leak. Interestingly, this robust effect of statins on spark characteristics was lost following fiber permeabilization (Supplemental Figure S1), which explains discrepancies with previous work 20, 21, 23 and suggests that statin effects in intact cells depend on the normal regulation of the RyR1 and/or effects of a soluble mediator.

An important question is whether statin-induced SR Ca^2+^ leak is also seen in cardiac muscle because this could have additional detrimental consequences by promoting triggered arrhythmias (40). Reassuringly, there was minimal impact of statin treatment on Ca^2+^ sparks in intact cardiac myocytes from statin-treated rats (Figures 2D to 2F). Thus, statin treatment induces SR Ca^2+^ leak in skeletal muscle, whereas cardiac muscle is protected from this potentially deleterious effect.

### NOS and reactive oxygen species promote SR Ca^2+^ leak with statin treatment

**Figure 3:**
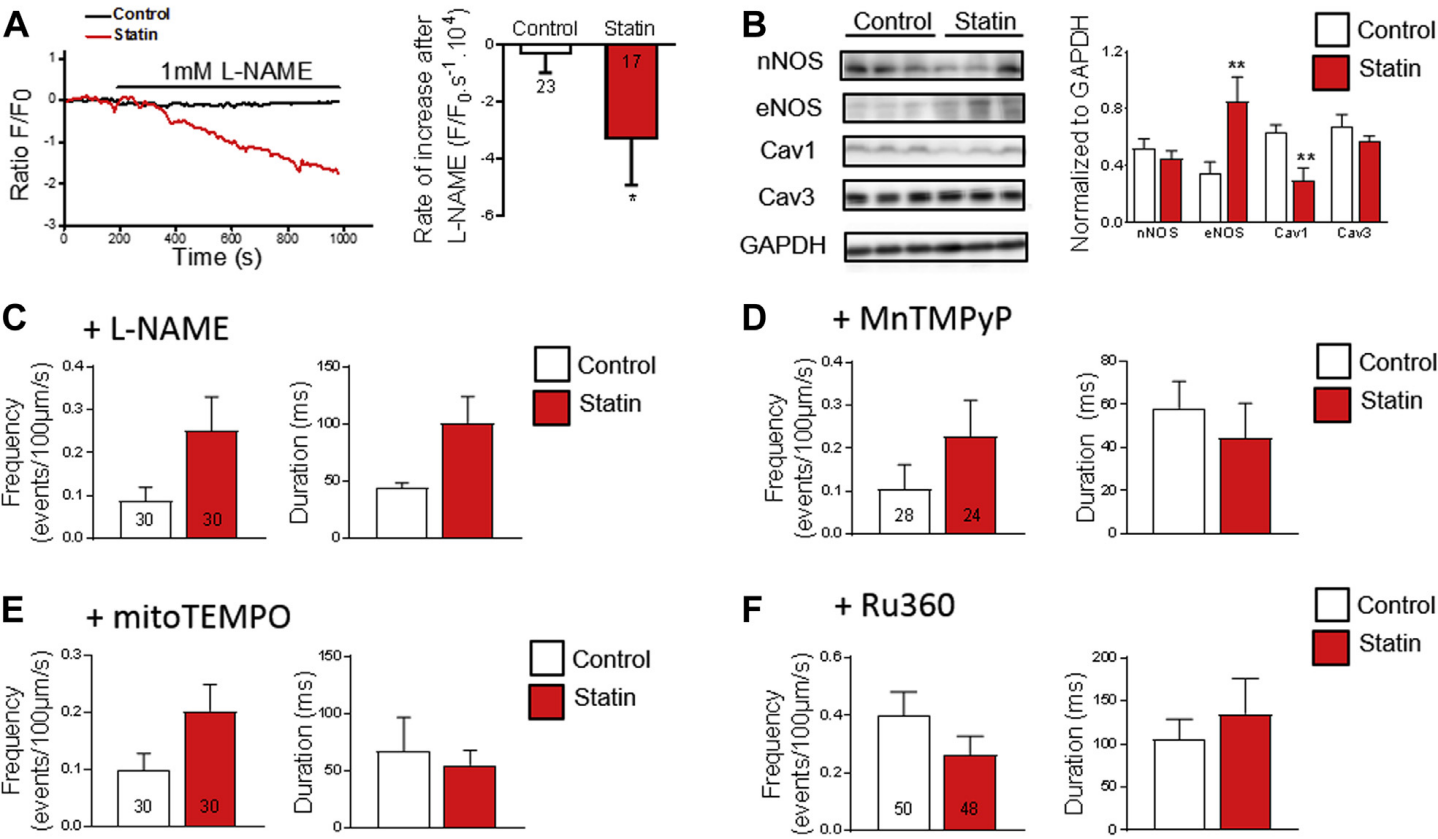
Nitric Oxide Synthase and Reactive Oxygen Species Promote SR Ca^2+^ Leak With Statin Treatment **(A)** Representative traces and mean data showing the impact of N(ω)-nitro-L-arginine methyl ester (L-NAME) (1 mM) on DAF-2 fluorescence in FDB fibers. Within each cell, the rate of increase in DAF-2 fluorescence recorded before L-NAME was subtracted from the entire trace; n = 7/8 animals for control/statin-treated rats, number of cells shown on graph. **(B)** Representative blots from the same gel and mean data from rat gastrocnemius (n = 9 to 11 animals). Ca^2+^ spark frequency and duration in intact FDB fibers in the presence of **(C)** L-NAME (1 mM), **(D)** Mn (III)tetrakis(1-methyl-4-pyridyl)porphyrin (MnTMPyP) (0.1 mM), and **(E)** (2-(2,2,6,6-Tetramethylpiperidin-1-oxyl-4-ylamino)-2-oxoethyl)triphenylphosphonium chloride (mitoTEMPO) (25 μM); n = 3 animals, number of cells shown on graphs. **(F)** The impact of the mitochondrial Ca^2+^ uniporter inhibitor Ru360 (20 μM, 15 min) in intact FDB fibers on increased Ca^2+^ spark frequency and duration. Data from 7 animals (number of cells shown on graph). All data are mean + SEM and compared using Mann-Whitney rank test. **(A)** * p = 0.034. **(B)** ** p = 0.0042 (endothelial nitric oxygen synthase [eNOS]), ∗∗p = 0.0073 (caveolin 1 [Cav1]); other abbreviations as in Figures 1 and 2.

Reactive nitrogen species and reactive oxygen species (RNS/ROS) could account for the statin-induced SR Ca^2+^ leak; both can be increased by statin treatment 25, 41, 42, target the RyR and its associated proteins directly 43, 44 and indirectly (45), and affect RyR activity (46). Inhibition of NOS isoforms with N(ω)-nitro-L-arginine methyl ester (L-NAME) had a greater impact on NO (indexed with DAF-2) in FDB fibers from statin-treated rats than in control rats, which was consistent with higher NOS activity in the statin group (Figure 3A). This could be explained by increased expression of endothelial NOS and reduced expression of the NOS-inhibitory caveolin isoform Cav1 (Figure 3B). These observations were consistent with statins acting as inhibitors of HMG CoA reductase and established pathways in which products of the HMG CoA reductase cascade regulate NOS (isoprenoids) and caveolin (cholesterol) expression (47). Enhanced NOS activity was directly linked with Ca^2+^ leak because, in the presence of L-NAME, there was no longer any difference (p > 0.05) in Ca^2+^ spark frequency or duration between fibers from control and statin-treated rats (Figure 3C). L-NAME inhibits NO and superoxide production from NOS (48), which indicates a role for NO, superoxide, and/or peroxynitrite in the spark-mediated leak.

Statin treatment has been shown to increase ROS production in skeletal muscle (25). We showed that these ROS played a role in the SR Ca^2+^ leak, because the superoxide dismutase (SOD) and peroxynitrite scavenger Mn(III)tetrakis(1-methyl-4-pyridyl)porphyrin (MnTMPyP) and the mitochondrial-targeted SOD mimetic (2-(2,2,6,6-Tetramethylpiperidin-1-oxyl-4-ylamino)-2-oxoethyl)triphenylphosphonium chloride (mitoTEMPO) eliminated the difference (p > 0.05) in Ca^2+^ spark frequency and duration between fibers from control and statin-treated rats (Figures 3D and 3E).

Bidirectional Ca^2+^ fluxes between SR and mitochondria affect both SR and mitochondrial function. Mitochondria accumulate close to SR Ca^2+^ release sites during postnatal skeletal muscle maturation, which facilitates mitochondrial Ca^2+^ uptake and is associated with reduced susceptibility to Ca^2+^ spark activation (49). Conversely, excessive mitochondrial Ca^2+^ uptake may promote Ca^2+^ sparks by enhancing ROS production from complexes I and III 50, 51. In support of this latter mechanism, the difference in Ca^2+^ spark frequency and duration between fibers from control and statin-treated rats was no longer present after inhibiting Ca^2+^ entry into the mitochondria via the mitochondrial Ca^2+^ uniporter with Ru360 (52) (Figure 3F). Taken together, the impact of NOS inhibition with L-NAME, ROS scavengers, and mitochondrial Ca^2+^ uniporter inhibition suggests that mitochondrial Ca^2+^ uptake stimulates RNS/ROS production, which, in turn, acts on RyR1 to maintain and/or exacerbate the SR Ca^2+^ leak.

### Consequences of statin-induced SR Ca^2+^ leak for muscle function

**Figure 4:**
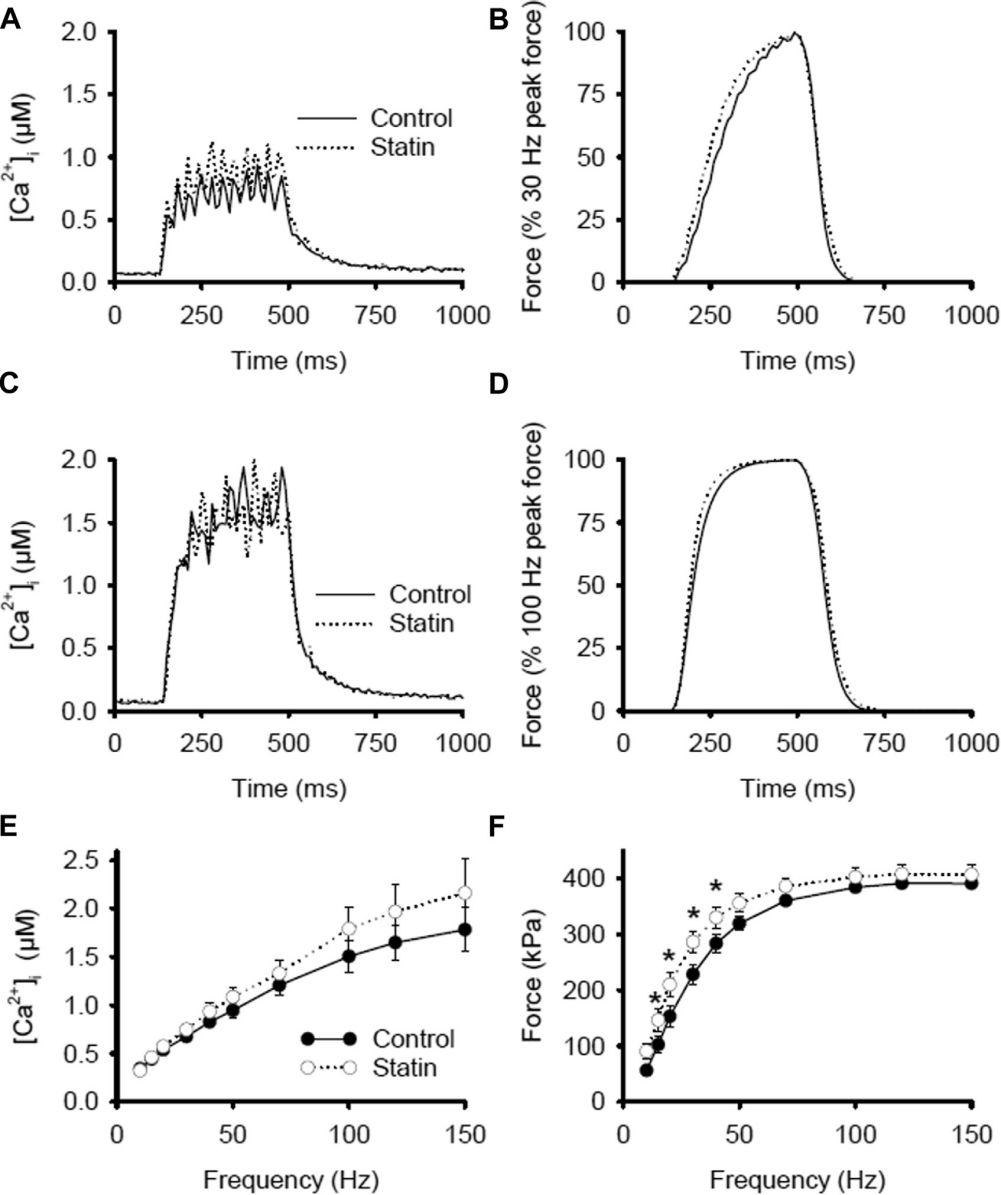
Impact of Statin Treatment on Basal and Tetanic [Ca^2+^]_i_ and Force Production Representative [Ca^2+^]_i_ transients at **(A)** 30 Hz and **(C)** 100 Hz, and **(E)** mean data for 10 to 150 Hz in FDB fibers stimulated with 350 ms trains of pulses at 1-min intervals. Corresponding representative force records at **(B)** 30 Hz and **(D)** 100 Hz, and **(F)** mean data for 10 to 150 Hz. From n = 13/11 fibers from 5/6 animals for control/statin-treated animals. Data are mean ± SEM and compared with 2-way repeated-measures analysis of variance. **(F)** *p = 0.020 (15 Hz); p = 0.003 (20 Hz); p = 0.005 (30 Hz); p = 0.042 (40 Hz) versus control subjects. Abbreviations as in Figure 2.

Next, we determined whether the observed effects of statins had a net impact on muscle function by measuring the free cytosolic [Ca^2+^] ([Ca^2+^]_i_) and force production in electrically stimulated single FDB fibers (30). There was no significant difference in basal [Ca^2+^]_i_ (69 ± 5 nM vs. 72 ± 3 nM; n = 13/11) or tetanic [Ca^2+^]_i_ (Figures 4A, 4C, and 4E) between FDB fibers from control and statin-treated animals. Furthermore, statin treatment did not reduce force production at any frequency; at low-frequency stimulation (≤40 Hz) there was a small increase in tetanic force in the statin group (Figures 4B and 4F). Calpains belong to a family of Ca^2+^-dependent proteolytic enzymes with pro-apoptotic activity (53). Calpain activity did not differ between control and statin-treated muscle (9.3 ± 0.4 vs. 9.3 ± 0.2 AU; n = 10), which was consistent with the unaltered basal [Ca^2+^]_i_. Thus, in resting muscle, the statin-induced SR Ca^2+^ leak was effectively counteracted by alterations in SR Ca^2+^ uptake and/or Ca^2+^ fluxes across the cell membrane (54). This concept of compensated leak was proposed to explain normal basal [Ca^2+^]_i_ in conjunction with Ca^2+^ leak from RyR1 due to a mutation found in malignant hyperthermia (Y522S) (36). During contractions, the amount of Ca^2+^ released in response to action potential stimulation remains constant over a wide range of SR Ca^2+^ content in fast-twitch muscle fibers (55), and depletion of SR Ca^2+^ promotes refilling via store-operated Ca^2+^ entry (56). Thus, the unaffected tetanic [Ca^2+^]_i_ in muscle fibers from statin-treated rats was also compatible with the observed spark-mediated SR Ca^2+^ leak in these fibers.

### Moderate exercise reverses the impact of statins on skeletal muscle

**Figure 5:**
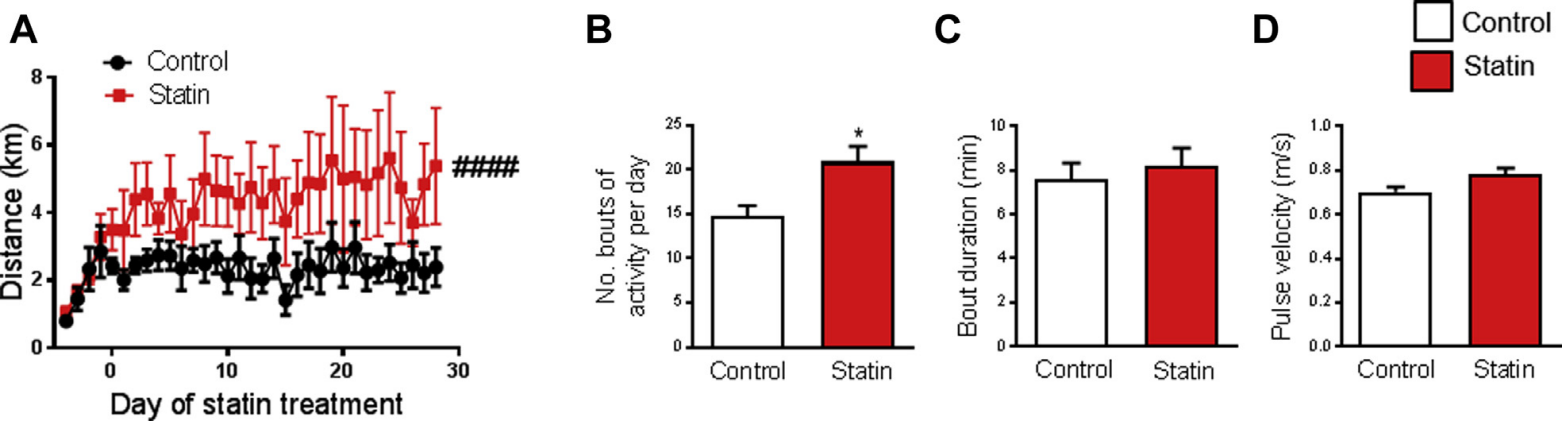
Statin Treatment Increases Physical Activity Running wheel data from the dark cycle (active period). Rats were acclimatized to the wheel for 4 days before statin treatment commenced (day 0). **(A)** Daily running distance over the duration of the study. **(B to D)** Detailed analysis of activity for the 28-day treatment period. Bouts (continuous periods of activity) were defined as activity seen in ≥2 consecutive minutes. Pulse velocity represents the mean velocity of each one-quarter revolution (pulse) of the wheel. Data are mean ± SEM (n = 6 animals per group). **(A)** ####p < 0.0001 for statin effect (2-way analysis of variance). **(B)** *p = 0.0148 versus control (*t*-test).

Exercise is recommended for those at risk of cardiovascular disease (i.e., those who take statins). However, there are reports that exercise reveals or exacerbates statin myalgia 13, 14 and myositis 12, 15, 16, and that statins limit training adaptations in skeletal muscle 57, 58. Therefore, we gave statin-treated and control rats access to an in-cage running wheel, which resulted in a type of voluntary exercise similar to that recommended for human subjects prescribed statins. Rats were acclimatized to the wheel for 4 days before statin treatment commenced. Unexpectedly, the daily running distance was greater for statin-treated rats than for control rats across the 4 weeks of the study (Figure 5A). The larger daily running distance in the statin group was due to an increase in the number of bouts of activity (Figure 5B), whereas the running bout duration (Figure 5C) and running velocity (Figure 5D) were similar in the 2 groups.

**Figure 6:**
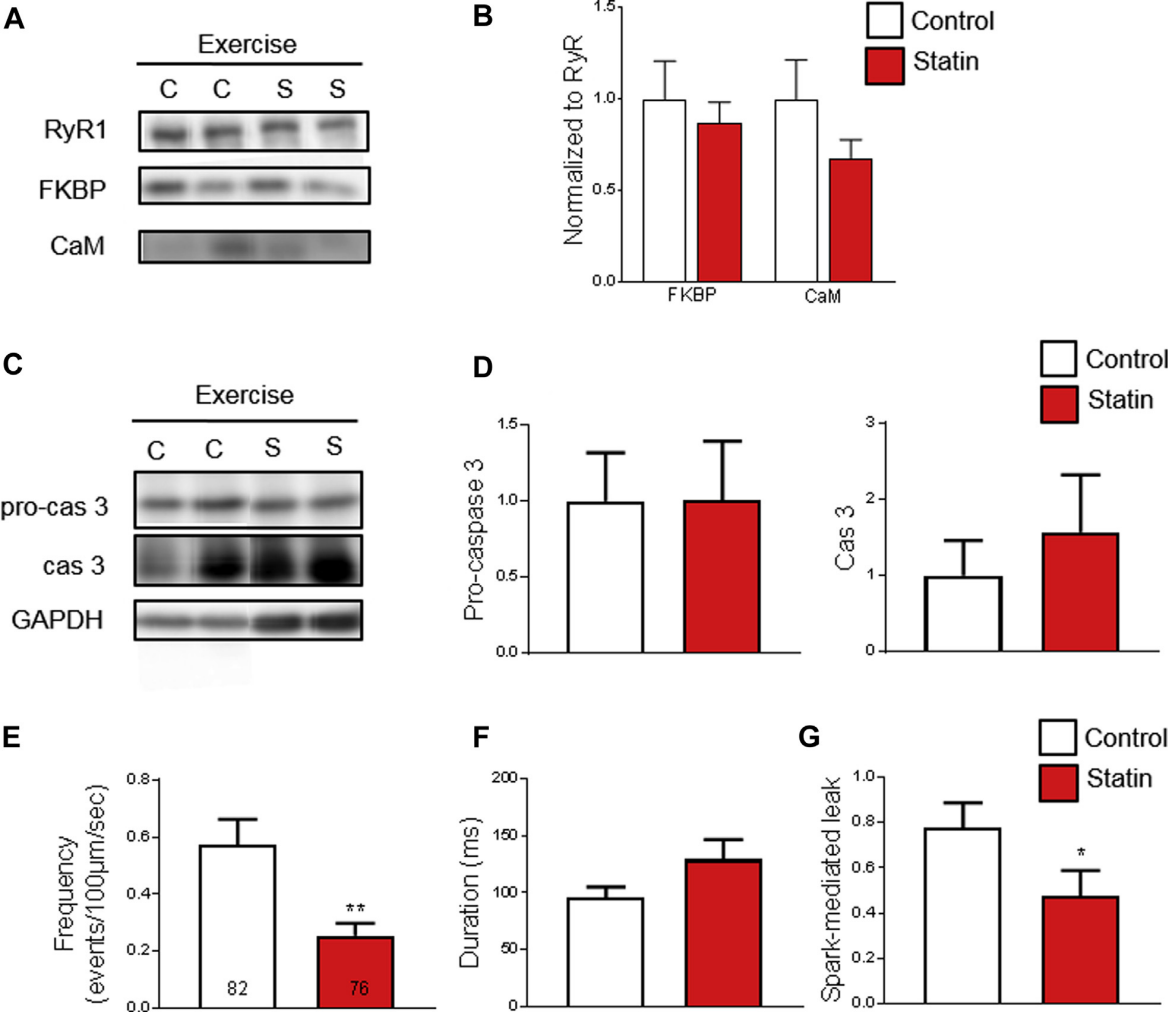
Exercise Reverses the Effect of Statin Treatment on the RyR Complex, Apoptosis, and SR Ca^2+^ Leak **(A)** Representative blots and **(B)** mean data of FKBP12 and CaM in RyR immunoprecipitates from gastrocnemius muscle of exercised animals. Data are normalized to RyR and standardized to the mean of the control exercise group (n = 6 animals). C = control, S = statin. **(C)** Representative blots and **(D)** mean data of pro-cas 3 and cleaved cas 3 expression in gastrocnemius homogenates of exercised animals. Data are normalized to GAPDH and standardized to the mean of the control exercise group (n = 6 animals). **(E)** Mean data for spark frequency, duration, and spark-mediated leak in intact FDB fibers from n = 6 exercised animals per group, number of cells shown on graph. Data are mean + SEM and compared using the **(B)** Student’s *t*-test and **(D to G)** Mann-Whitney. *p = 0.0197; **p = 0.0061. Abbreviations as in Figures 1, 2, and 3.

In sharp contrast to the situation in muscles of sedentary subjects (see Figure 1), binding of FKBP12 to RyR1 showed no significant difference between muscles of statin-treated and control rats after 4 weeks of exercise (Figures 6A and 6B). Moreover, in the exercised state, statin treatment no longer caused a significant increase in caspase 3 expression (Figures 6C and 6D). Intriguingly, in the exercised state, the frequency of SR Ca^2+^ sparks was lower in muscle fibers of statin-treated rats than in control rats, which contributed to a smaller spark-mediated Ca^2+^ leak in this group (Figures 6E to 6G).

**Figure 7:**
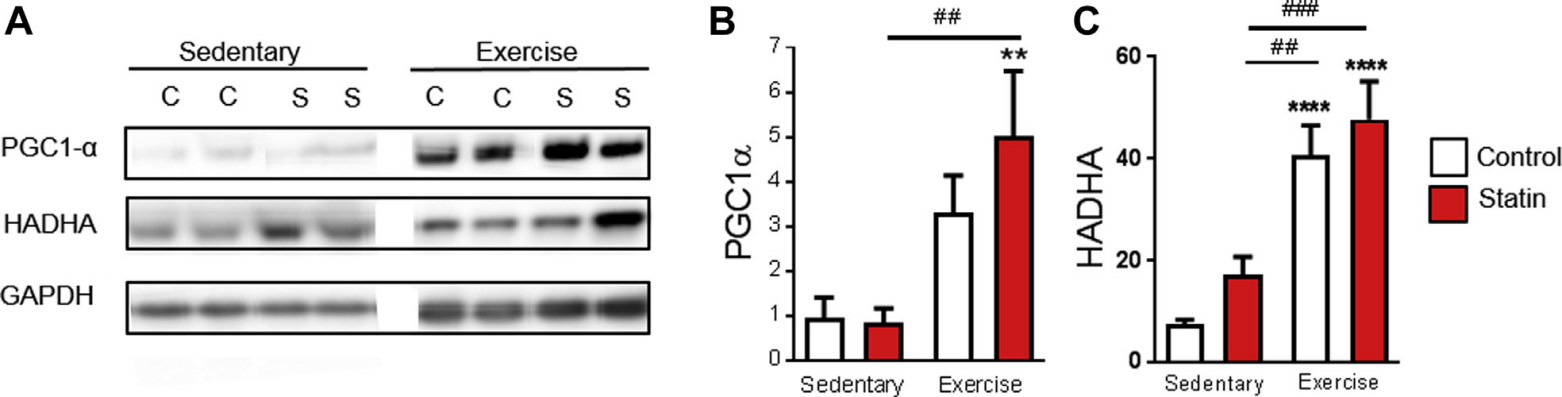
Statins Do Not Limit Mitochondrial Biogenesis in Sedentary or Exercised Rats **(A)** Representative blots and **(B and C)** mean data from rat gastrocnemius homogenates showing peroxisome proliferator-activated receptor γ co-activator 1α (PGC1α) and hydroxyacyl-CoA dehydrogenase/3-ketoacyl-CoA thiolase/enoyl-CoA hydratase (HADHA) expression. Mean data are normalized to GAPDH. Data are mean + SEM from 6 to 11 animals. **(B)** **p = 0.0034 versus sedentary control animals; ##p = 0.0034 between groups as indicated. **(C)** ****p < 0.0001 versus sedentary control animals; ##p = 0.0025; ###p = 0.0001 between groups as indicated (2-way analysis of variance with Tukey test for post hoc analysis). Abbreviation as in Figure 1.

A number of studies have linked statin-induced myopathy with impaired mitochondrial biogenesis. The transcriptional co-activator PGC1 is a key mediator of mitochondrial biogenesis in response to endurance exercise 59, 60, 61. Statin treatment has been shown to decrease PGC1α mRNA expression in human and rodent fast skeletal muscle (24); however, no change in PGC1α protein was detected in rodent muscle (62). We saw no impact of statin treatment on protein expression of PGC1α or HADHA (which is used as an index of mitochondrial biogenesis) either in sedentary or exercised animals (Figures 7A to 7C). Together, these data show that statin treatment did not limit moderate physical activity or markers of training adaptation in skeletal muscle. Exercise reversed the statin-dependent SR Ca^2+^ leak, which suggests a potentially beneficial effect.

## Discussion

The prevalence of statin-induced muscle symptoms varies between 7% and 29% in registries and observational studies (63). Thus, most patients taking statins do not experience skeletal muscle problems. In skeletal muscle of statin-treated humans and rats, we showed FKBP12 dissociation from RyR1, which resulted in a ROS/RNS−dependent Ca^2+^ spark-mediated SR Ca^2+^ leak. Such destabilization of RyR1 has been associated with muscle dysfunction in a variety of conditions, including heart failure, aging, and muscular dystrophy 33, 34, 35. Accordingly, we observed indexes of pro-apoptotic signaling in statin-treated subjects. Nevertheless, in the rodent model, statin-induced FKBP12 dissociation from RyR1 and Ca^2+^ sparks were not accompanied by any obvious defects in the overall control of [Ca^2+^]_i_ at rest or during tetanic stimulation, and force production was not decreased. Unaltered muscle function, despite potentially deleterious changes in cellular Ca^2+^ handling, fits with the clinical picture that although statin treatment increases the risk of myopathy, most patients do not experience statin-associated adverse muscle symptoms. Indeed, analysis of mitochondrial DNA and muscle gene expression profiles in a small group of patients taking simvastatin for 8 weeks revealed evidence of mitochondrial damage, pro-apoptotic signaling, and altered Ca^2+^ flux despite an absence of muscle symptoms (64). Thus, we argue that statin treatment initiates potentially detrimental changes in skeletal muscle as a result of Ca^2+^ dysregulation, but that this does not usually translate into myalgia or more serious muscle derangement.

### Cardiac muscle is protected from statin-induced myopathy

In contrast to skeletal muscle, we observed no increase in spark-mediated SR Ca^2+^ leak in cardiac myocytes from statin-treated rats. Our results showed a central role of increased ROS/RNS in the statin-induced destabilization of SR Ca^2+^ control. This offers a simple explanation for the selectivity of statins for skeletal muscle: cardiac muscle has superior enzymatic (e.g., SOD) and nonenzymatic (e.g., glutathione) ROS/RNS defense systems to fast skeletal muscle 24, 25. Furthermore, statin treatment has been shown to enhance the antioxidant defense in cardiac muscle while limiting the defense in skeletal muscle (24). In addition, direct effects of the statin molecule on RyR might also contribute to selective skeletal myopathy. In planar lipid bilayers, simvastatin increases the open probability of RyR1 but not RyR2 (28). Similarly, acute application of simvastatin to permeabilized cells shifts the distribution of Ca^2+^ spark frequency toward higher values in skeletal fibers (which express predominantly RyR1) but lower values in cardiac myocytes (which express RyR2) (28). Thus, a central role for RyR in statin myopathy fully explains the selectivity of this effect for skeletal over cardiac muscle.

### Moderate exercise may limit deterimental effects of statins on skeletal muscle

The prevalence of statin-induced myopathy has been reported to increase with physical activity in rodent models (16) and in humans 12, 15. RNS/ROS increase during exercise and strenuous skeletal muscle activity can result in severe FKBP12 dissociation from RyR1 and impaired contractile function 65, 66, thus providing a mechanism by which exercise could exaggerate the negative effects of statin treatment. In contrast, increased RNS/ROS and altered SR Ca^2+^ handling play an important role in the adaptation to endurance training 67, 68, 69, 70, and a moderate SR Ca^2+^ leak has been linked to increased fatigue resistance 71, 72. We showed beneficial effects of voluntary running exercise in muscle of statin-treated rats. Statin treatment no longer reduced FKBP12 binding to RyR1, increased caspase 3 expression, or increased Ca^2+^ spark frequency. Measures of mitochondrial biogenesis (PGC1α and HADHA expression) were enhanced, at least to the same extent, as in muscle of trained control rats. Thus, our results imply that combining moderate voluntary exercise with statin treatment is not detrimental and might limit potentially harmful muscle effects of statins. Of note, most reports of exacerbation of statin myopathy are with intense, prolonged, or enforced exercise regimens 12, 15, 16. Data from the PRIMO study (73) hinted at the relationship between exercise intensity and the incidence of statin-associated muscle symptoms, and it was recently suggested that reducing the intensity of exercise could mitigate the myopathy risk (18). The opposing intensity-dependent effects of exercise likely reflect a narrow span between limited FKBP12 dissociation from RyR1 accompanied by improved muscle endurance 71, 72 and severe FKBP12 dissociation resembling ‘overtraining’ with marked muscle weakness 65, 66.

Unexpectedly, statin-treated rats performed more bouts of activity per day, which translated into longer distances run, compared with control rats. This finding seemingly excluded statin-induced muscle pain or other sensory-related symptoms, because such symptoms were unlikely to result in an increased willingness to perform voluntary exercise. The increased voluntary running of statin-treated rats might relate to their increased muscular NO production, because mice given dietary nitrate supplementation run more than control mice (74).

### Study limitations and significance

A limitation of the present study is that although we identified a potentially harmful effect of statin treatment, we did not provide direct evidence of conditions in which the increased SR Ca^2+^ leak resulted in myopathic symptoms. The likely scenario is that the statin-induced RyR1 destabilization has to be combined with other factor(s) for myopathic symptoms to occur. The concept that individuals might be genetically predisposed to myopathy as a result of altered statin metabolism and/or muscle susceptibility is gaining acceptance 75, 76, 77. There is strong support for dysregulation of Ca^2+^ handling contributing to muscle susceptibility. For example, disease-causing mutations or rare variants in *RyR1* have been found in those who experienced statin-associated muscle symptoms (78). Nearly one-fifth of a cohort of subjects who had severe statin myositis had rare variants within genes for RyR1 and the pore-forming subunit of the L-type Ca^2+^ channel (76). Gene expression analysis of muscle from patients with a history of statin myalgia who were re-challenged with statins revealed a number of pathways and networks linked with RyR regulatory proteins, including calmodulin and autocrine motility factor (which plays a role in endoplasmic reticulum (ER)/SR-mitochondrial communication) (79), and regulatory Ca^2+^-binding proteins (calpain, calcineurin) (75). Accordingly, in a study on patients with statin-induced myositis, most (7 of 9) of the in vitro muscle tests showed halothane- and caffeine-induced contractures suggestive of impaired SR Ca^2+^ control and, in 1 patient, the abnormality was consistent with malignant hyperthermia, a disorder linked to variants in *RYR1* (80). Moreover, lifestyle habits, such as excessive exercise regimens that induce SR Ca^2+^ leak via RyR1 FKBP12 dissociation, might also reveal overt myopathy with statin treatment as discussed above.

We only studied rats treated with statins for 4 weeks, and it is possible that a more prolonged treatment could result in functional abnormalities in muscle. However, because statin myopathy can occur at any time during long-term statin treatment (81) and most people taking statins over many years do not experience overt muscle symptoms, this suggests that this is generally not the case. Thus, it is an additional susceptibility (e.g., genetic or exercise-induced SR Ca2+ leak) that reveals myopathy in a small proportion of the cohort.

## Conclusions

Conditions in which increased SR Ca^2+^ leak can be expected should be considered a risk factor when statins are prescribed. Identifying risk factors underlying statin-induced myopathy is important because recent modeling experiments have indicated that improving statin adherence by 50% (e.g., by preventing statin-induced myopathy) would prevent twice as many deaths as a 5% reduction in the cardiovascular risk threshold for statin prescription (82)*.*

Perspectives **COMPETENCY IN MEDICAL KNOWLEDGE:** Up to one-third of patients report statin-associated muscle symptoms in observational studies. The incidence in randomized controlled trials is much less. In part, this difference may arise because those susceptible to myopathy or with indications of myopathy in the run-in phase are excluded from trials. However, the experience of muscle pain is subjective, and many patients are primed to expect this because of patient information leaflets and widespread reporting of side effects of statins in the press. Therefore, an understanding of the mechanism of statin myopathy and factors that make users more susceptible to overt muscle pain and weakness (even potentially fatal rhabdomyolysis) are essential. In this study, we demonstrated leaky RyRs in skeletal muscle following statin treatment. Although this by itself did not cause overt myopathy, it did provide a strong indicator of the populations who are at real risk of myopathy—those whose lifestyle or genotype predispose them to SR Ca^2+^ leak. This includes patients who undertake regular high-intensity exercise or have mutations in the RyR1 associated with malignant hyperthermia. In these individuals, statins should be used cautiously with consideration of dose, alternative cholesterol-lowering strategies, and monitoring of serum creatine kinase levels. However, our data do support the view that moderate exercise should be actively encouraged in those who take statins. As well as the positive effects of exercise on cardiovascular health, this type of activity appears to limit potentially harmful effects of statins on skeletal muscle.

**TRANSLATIONAL OUTLOOK:** There are several barriers to clinical translation of this work. The first is the sheer scale of the problem, because of the number of people who are (and should be) prescribed statins. Second, we have not yet identified directly the conditions that precipitate overt myopathy, although our data provided a strong indication of what these factors may be. Third, there are currently no cost-effective alternative antilipidemic agents that match the efficacy of statins for those at high risk of myopathy. Statins confer additional therapeutic benefits independent of their ability to lower serum cholesterol (pleiotropic actions), which are not evident with other drugs. For example, the recently licensed PCSK9 inhibitors cost 50 to 100 times more than generic statins and lack the pleiotropic actions effects of statins.
