# Supplementary material for: A Mechanism for Statin-Induced Susceptibility to Myopathy
Source: JACC Basic Transl Sci. 2019 Aug 26;4(4):509–23. doi: 10.1016/j.jacbts.2019.03.012 (PMC6712048; doi:10.1016/j.jacbts.2019.03.012)
Supplement: Supplemental Data [file mmc1.pdf]

Supplemental Material for:

A mechanism for statin-induced susceptibility to myopathy

Sabine Lotteau, Niklas Ivarsson, Zhaokang Yang, Damien Restagno, John Colyer, Philip Hopkins, Andrew Weightman, Koichi Himori, Takashi Yamada, Joseph Bruton, Derek Steele, Håkan Westerblad, Sarah Calaghan

METHODS

STATIN DOSE: 40 mg/kg/day of simvastatin gives clinically relevant peak plasma levels between 5-10 times the IC_{50} in this species, see (1, 2). The dose is higher than that used in man (up to 80 mg daily; i.e. 1.1 mg/kg/day for a 70 kg patient). However, simple scaling for bodyweight is inadequate to convert rodent doses to human equivalents. Allometric scaling, based on body surface area, is recommended by the Food and Drug Administration (3) and used in the pharmaceutical industry (4); using this approach 40 mg/kg in rat equates to a 400 mg dose in man (5), which is only 5 times higher than the maximum prescribed human dose (80 mg). Furthermore, additional scaling factors should be applied because simvastatin is highly metabolized and undergoes biliary excretion (4) further reducing the equivalent human dose. Importantly, our data show the same effect of statin treatment in rodent muscle as in muscles from patients taking statins, providing support for our choice of simvastatin dose in the rat.

EXERCISE ANALYSIS. All data shown are from the dark cycle; there was minimal activity in all animals during the light cycle. A rat was considered to be active when there were ≥4 revolutions of the running wheel during each 1 min recording period. This equates to ≈ 10% of the mean dark cycle velocity of running for control animals (0.69 m/s). Continuous periods of activity (bouts) were defined as activity seen in 2 or more consecutive minutes.

MUSCLE FUNCTION IN VITRO. The indo-1 fluorescence was converted to free myoplasmic $[Ca^{2+}]$ ($[Ca^{2+}]_i$) (6). In FDB fibers, basal $[Ca^{2+}]_i$ was measured from the mean indo-1 fluorescence over ~200 ms immediately prior to tetanic stimulation. Tetanic $[Ca^{2+}]_i$ was measured from the mean indo-1 fluorescence during the tetanic stimulation trains. Tetanic force was measured as the mean force over the final 100 ms of the contraction.

CONFOCAL MICROSCOPY. Events with a full width at half maximum (FWHM) < 1 µm were filtered from the data. Spark mass was calculated as amplitude x 1.206 x $FWHM^3$ and spark-mediated leak as spark mass x spark frequency (7). Ca^{2+} sparks in intact FDB and cardiac myocytes were recorded in the absence of electrical stimulation. All experiments were performed with air-equilibrated solutions. The pO_2 of air is ≈160 mm Hg, hence higher than that of arterial blood (80-100 mm Hg) and this could

create oxidative stress. However, ROS scavengers had no effect on Ca^{2+} spark frequency or duration in intact FDB fibers from control animals, suggesting minimal oxidative stress under our experimental conditions, in the absence of statin treatment.

IMMUNOPRECIPITATION. Muscle was homogenized in homogenization buffer containing (mM): HEPES 20, NaCl 150, EDTA 5, KF 25, Na-orthovanadate 1 with glycerol 20%, triton X-100 0.5% and protease inhibitor cocktail (Roche, cOmplete), pH 6.8. Anti-RyR1 antibody (#2868, Abcam) was incubated with Dynabeads Protein G (Invitrogen) for 40 min at room temperature (1 µl antibody + 11 µl beads per sample). The beads were washed with PBS-0.05% Tween 20 twice and homogenization buffer once. An aliquot (400 µl) of lysate (total protein concentration of 1.5 mg/ml or 1 mg/ml for human and rat muscle respectively) was incubated at 4°C overnight with antibody-bead slurry in homogenisation buffer. After washing, proteins were separated on SDS-PAGE and Western blotting performed. Data were normalized to RyR1 in the immunoprecipitated sample. For human samples, RyR1 pulldown showed more inter-sample variability than in rat, however RyR1 was undetectable in the voided fractions.

RESULTS

TABLE S1: Summary of experiments performed and key findings

Experiment	Human	Sedentary rat	Exercised rat
RyR1 complex	↓ FKBP<br>→ CaM	↓ FKBP<br>→ CaM	→ FKBP<br>→ CaM
Pro-apoptotic signalling (caspase)	↑ Cas3	↑ Cas3	→ Cas3
Pro-apoptotic signalling (TUNEL)		↑ TUNEL positive nuclei	
Ca^{2+} sparks (intact skeletal fibres)		↑ frequency<br>↑ duration<br>↑ amplitude<br>↑ mass<br>↑ leak	↓ frequency<br>→ duration<br>↓ leak
Ca^{2+} sparks (permeablized skeletal fibres)		→ frequency<br>→ duration<br>→ amplitude<br>→ mass<br>→ leak	
Ca^{2+} sparks (intact cardiac myocytes)		→ frequency<br>↑ duration<br>→ amplitude<br>→ mass<br>→ leak	
+ NOS inhibition (DAF2 fluorescence)		↓ DAF2	
+ NOS inhibition (Ca^{2+} sparks)		→ frequency<br>→ duration	
NOS regulatory protein expression		↑ eNOS<br>→ nNOS<br>↓ caveolin 1<br>→ caveolin 3	
+ ROS scavenging (MnTMPyP) (Ca^{2+} sparks)		→ frequency<br>→ duration	
+ ROS scavenging (mitoTEMPO) (Ca^{2+} sparks)		→ frequency<br>→ duration	
+ MCU inhibition (Ca^{2+} sparks)		→ frequency<br>→ duration	
$[Ca^{2+}]_i$		→ basal<br>→ tetanic	
Force		→ tetanic (> 40 Hz)	
Calpain		→ activity	
Running wheel activity			↑ distance<br>↑ bouts activity<br>→ bout duration<br>→ velocity

Mitochondrial biogenesis		→ PGC1α<br>→ HADHA	→ PGC1α<br>→ HADHA

Data in the table show the effect of statin treatment in human and rat skeletal muscle (unless stated otherwise). ↑ ↓ → increased, decreased or unchanged with statin treatment respectively. FKBP, FK506 - binding protein; CaM, calmodulin; Cas3, cleaved caspase 3; NOS, nitric oxide synthase; MnTMPyP, Mn(III)tetrakis(1-methyl-4-pyridyl)porphyrin; MitoTEMPO, (2-(2,2,6,6-Tetramethylpiperidin-1-oxyl-4-ylamino)-2-oxoethyl)triphenylphosphonium chloride; MCU, mitochondrial calcium uniporter; PGC1α, peroxisome proliferator-activated receptor γ co-activator 1; HADHA, hydroxyacyl-CoA dehydrogenase/3-ketoacyl-CoA thiolase/enoyl-CoA hydratase.

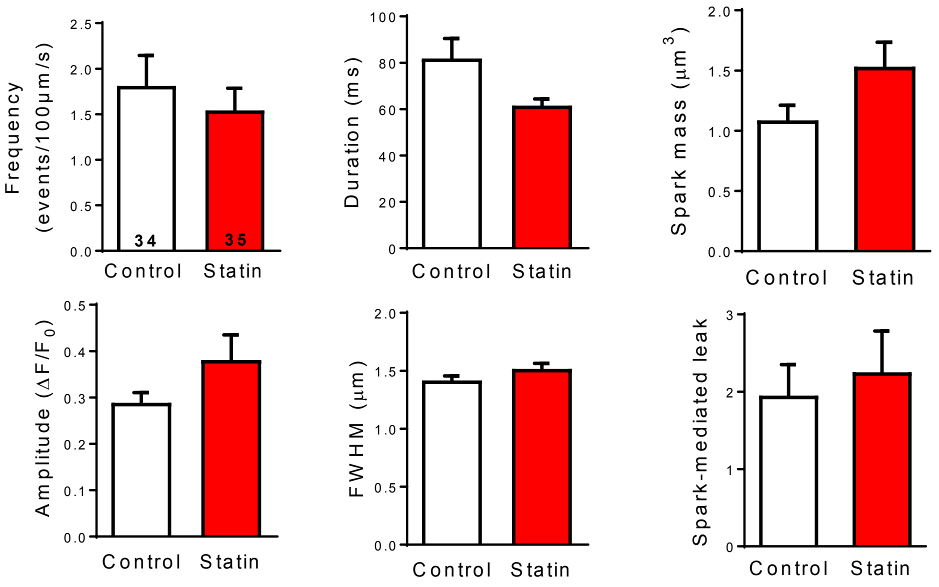

FIGURE S1. The effect of statin treatment on Ca^{2+} spark parameters in saponin-permeabilized skeletal muscle fibers (FDB). Note that spark frequency is >5 times higher in permeabilized fibers compared with intact fibers. FWHM, full width at half maximum. Data are mean + S.E.M. for n=8-9 animals (number of cells shown on graph; Mann-Whitney rank test).
